# Supplementary material for: Multi‐dataset identification of innovative feature genes and molecular mechanisms in keratoconus
Source: J Cell Mol Med. 2024 Sep 19;28(18):e70079. doi: 10.1111/jcmm.70079 (PMC11412914; doi:10.1111/jcmm.70079)
Supplement: Supplementary file 2 — Material S2. [file JCMM-28-e70079-s002.zip › 7.GSEA analysis/4.MYRF/KEGG_DORSO_VENTRAL_AXIS_FORMATION.html]

Details for gene set KEGG\_DORSO\_VENTRAL\_AXIS\_FORMATION[GSEA]

|  || Dataset | MYRF.group.cls#h\_versus\_l |
| Phenotype | group.cls#h\_versus\_l |
| Upregulated in class | l |
| GeneSet | KEGG\_DORSO\_VENTRAL\_AXIS\_FORMATION |
| Enrichment Score (ES) | -0.4356436 |
| Normalized Enrichment Score (NES) | -1.3667983 |
| Nominal p-value | 0.13104838 |
| FDR q-value | 0.25332132 |
| FWER p-Value | 0.912 |
Table: GSEA Results Summary

  

Fig 1: Enrichment plot: KEGG\_DORSO\_VENTRAL\_AXIS\_FORMATION      
 Profile of the Running ES Score & Positions of GeneSet Members on the Rank Ordered List

  

| SYMBOL | TITLE | RANK IN GENE LIST | RANK METRIC SCORE | RUNNING ES | CORE ENRICHMENT || 1 | CPEB1 | na | 4824 | 0.228 | -0.0718 | No |
| 2 | SOS1 | na | 6706 | 0.172 | -0.0751 | No |
| 3 | ETV6 | na | 7540 | 0.150 | -0.0550 | No |
| 4 | KRAS | na | 9371 | 0.108 | -0.0758 | No |
| 5 | NOTCH3 | na | 11451 | 0.071 | -0.1147 | No |
| 6 | SOS2 | na | 12783 | 0.053 | -0.1375 | No |
| 7 | PIWIL4 | na | 14188 | 0.036 | -0.1675 | No |
| 8 | MAPK3 | na | 14382 | 0.034 | -0.1631 | No |
| 9 | ETV7 | na | 15239 | 0.026 | -0.1802 | No |
| 10 | EGFR | na | 17031 | 0.008 | -0.2294 | No |
| 11 | NOTCH2 | na | 17277 | 0.006 | -0.2346 | No |
| 12 | PIWIL1 | na | 17657 | 0.003 | -0.2447 | No |
| 13 | MAPK1 | na | 20406 | -0.027 | -0.3161 | No |
| 14 | SPIRE1 | na | 20447 | -0.027 | -0.3093 | No |
| 15 | NOTCH1 | na | 23798 | -0.072 | -0.3845 | No |
| 16 | GRB2 | na | 24215 | -0.079 | -0.3731 | No |
| 17 | PIWIL3 | na | 24312 | -0.081 | -0.3520 | No |
| 18 | NOTCH4 | na | 27029 | -0.136 | -0.3902 | Yes |
| 19 | SPIRE2 | na | 27600 | -0.151 | -0.3622 | Yes |
| 20 | MAP2K1 | na | 30150 | -0.229 | -0.3681 | Yes |
| 21 | ETS2 | na | 30320 | -0.235 | -0.3038 | Yes |
| 22 | FMN2 | na | 32391 | -0.342 | -0.2625 | Yes |
| 23 | PIWIL2 | na | 33009 | -0.388 | -0.1658 | Yes |
| 24 | ETS1 | na | 34588 | -0.730 | 0.0040 | Yes |
Table: GSEA details [plain text format]

  

Fig 2: KEGG\_DORSO\_VENTRAL\_AXIS\_FORMATION      
 Blue-Pink O' Gram in the Space of the Analyzed GeneSet

  

Fig 3: KEGG\_DORSO\_VENTRAL\_AXIS\_FORMATION: Random ES distribution      
 Gene set null distribution of ES for **KEGG\_DORSO\_VENTRAL\_AXIS\_FORMATION**
